# Modeling of Residence Time Distributions of Twin-Screw Extrusion Processes Considering Various Screw Types

**DOI:** 10.3390/pharmaceutics18070883

**Published:** 2026-07-18

**Authors:** Vincent Kimmel, Dario Zöllig, Werner Hoheisel, Judith Winck, Markus Thommes

**Affiliations:** 1 Laboratory of Solids Process Engineering, Department of Biochemical and Chemical Engineering, Technical University Dortmund, Emil-Figge-Str. 68, 44227 Dortmund, Germany; vincent.kimmel@tu-dortmund.de (V.K.); dario.zoellig@tu-dortmund.de (D.Z.); judith.winck@tu-dortmund.de (J.W.); 2 INVITE GmbH, Drug Delivery Innovation Center, Chempark Building W32, Otto-Bayer-Str. 32, 51061 Cologne, Germany; 3 Bayer AG, 51368 Leverkusen, Germany; werner.hoheisel@bayer.com

**Keywords:** twin-screw extruder, screw characteristics, residence time distribution, mechanistic modeling

## Abstract

**Purpose**: Residence time in pharmaceutical hot-melt extrusion is a crucial process parameter affecting the quality attributes of a dosage form such as content uniformity, bioavailability and toxicity. There is a lack of knowledge about the influence of screw element types on the residence time distribution, which will be addressed in this study. **Methods**: Different conveying and kneading elements will be characterized concerning their effect on the residence time using a co-rotating twin-screw extruder with a 28 mm screw diameter. Tracer experiments will be performed while concentration time profiles are measured via inline UV-Vis spectroscopy. **Results**: The residence time distribution of all screw types is primarily related to the volume flow but independent of pressure. The two-compartment model of Reitz was utilized to describe the experimental data mathematically. Screw-type-specific dimensionless parameters were derived to quantify the axial mixing performance, as well as the effect on residence time. **Conclusions**: The influence of different screw elements on the residence time in hot-melt extrusion processes was quantified using a dimensionless mixing volume and a dimensionless axial dispersion coefficient. The results meet the quantitative descriptions from the literature.

## 1. Introduction

Hot-melt extrusion (HME) has been an emerging manufacturing technique in pharmaceutics for more than 30 years [1,2]. In accordance with the manufacturing classification system [3] it is grouped in class four as “other technologies”, having additional value in certain drug formulations. In this respect, it has been used for formulating poorly water-soluble drugs [4,5] and preparing modified release dosage forms [6], as well as special applications like flea collars [7] and insecticide-infused bed nets [8] to combat malaria. In these applications a crystalline drug substance is dissolved in a polymer melt at elevated temperatures while a mixing action is performed within the extruder [9]. Afterwards, the melt is cooled via a chill roll [10,11] or by calendaring [12], while the polymer transfers from the molten state to a glassy state in which the drug is molecularly dispersed. In terms of oral dosage forms, these systems are often called amorphous solid dispersions [13].

Co-rotating twin-screw extruders have been established in the pharmaceutical field [14] and have been widely used in the food and polymer industry for decades [15]. These machines have a high mixing performance with low equipment complexity [16], run continuously enabling quality by design [17], and are scaled by the screw diameter, allowing for a wide range of throughputs [18]. The two intermeshing screws are aligned parallel to one another in an extrusion barrel, which has a figure-eight-shaped bore. The screws consist of a sequence of screw elements of different geometry, which have different conveying and mixing performance characteristics. The sequence is adapted to the formulation properties, thereby tailoring the product to its specification [19].

In the preparation of amorphous solid dispersions, the formulation is transported axially through the extrusion barrel via the screws, while mechanical energy is transferred into heat and applied to the material (autogenic extrusion) [20]. This heat is utilized to melt the polymer and dissolve the drug substance. Based on this, there is a distinctive temperature profile in the axial direction (along the screws) after an equilibration period. Moreover, the material transport through the extruder is rather complex, since the material follows a distinctive flow pattern [21], having different residence times for similar volumes of material. This originates from differences in material velocity in the extruder even in the process equilibrium. Based on this, the time of temperature exposure (temperature–time profile) is not constant for subsets of material but can be described by a distribution function. Practically, each small volume element of material must experience enough temperature and heat for a certain period of time in order to dissolve all drug crystals in the polymer while processing. However, long residence times are harmful due to thermal degradation even if just a small fraction experiences these process conditions. This issue is particularly relevant for pharmaceutical applications, since high drug loads (>20% *w*/*w*) are usually used in an effort to reduce the "Pill Burden". This requires high temperatures, but temperature-induced degradation products do not meet the quality requirements.

In continuous manufacturing, the width of the residence time distribution is also related to the axial mixing performance of a process. Hence, wide residence time distributions are able to mitigate uniformity issues resulting from imperfections in material flow [22]. In twin-screw hot-melt extrusion, split feeding is quite common, where the different components such as the drug and excipient are fed separately into the extruder. This approach avoids an additional blending step and any segregation issues related to powder handling. However, the commonly used loss in weight feeders possess material-dependent fluctuations in the mass flow over time [15]. In this respect, wide residence time distributions are desirable to avoid content uniformity issues in the final product. But wide residence times, on the other hand, are also harmful since the traceability is hindered. The startup and equilibration of a process will require much more time, and when failure occurs, more material must be discarded. In this respect, the residence time distribution is a valuable tool to tailor the mixing performance of a process [23].

For formulation and process optimization, residence time measurements have become common in pharmaceutical applications for about the last 10 years [24]. To accomplish this, a tracer material is dropped at the inlet into an equilibrated extrusion process, and the tracer concentration is measured with respect to time in the final product [20]. In-line methods have been established to increase the precision of the measurements, reducing the experimental effort and allowing high sampling rates [25]. The obtained tracer concentration time profile ($c\left(t\right)$) is subsequently transferred into a probability density function ($E\left(t\right)$) by setting the integral of the $c\left(t\right)$ to 1 (Equation (1)). The integration is usually performed numerically using the trapezoid method [26]. The probability density function for a hot-melt extrusion process has a bell shape with a tailing to high residence times.

(1) $$E\left(t\right)=\frac{c\left(t\right)}{\int_{0}^{\infty }c\left(t\right)dt}$$

An integration of the probability density function leads to a probability function ($F\left(t\right)$, Equation (2)) having a sigmoidal shape [27]. Quantiles as statistical parameters have been utilized to explore these functions [28], where the onset is related to a minimal required dissolution time, and the offset connects to thermal degradation.

(2) $$F\left(t\right)=\int_{0}^{\infty }E\left(t\right)dt$$

Furthermore, mathematical models have been developed to describe residence time distribution behavior with the benefit of needing only a few parameters to describe the entire function. Residence models are particularly valuable in the determination of the onset and the offset of the distribution function because it is challenging to assess those by individual measurements. The conventional axial dispersion model [22], commonly used in process engineering, is not applicable to hot-melt extrusion processes. This model consists of only two parameters—the mean residence time as location parameter and the Bodenstein number as the spread parameter—and therefore cannot accurately represent the typical tailing of a residence time distribution (RTD) in an extrusion process [29]. Therefore, special models have been proposed (Table 1).

The early models were based on cascades of continuous stirred tank reactors by MacMullin and Weber [30], which were applied to extrusion processes by Kumar [34]. Janssen [31] has developed a model based on the leakage flow between the extruder screws and takes the screw geometry into account. The Zusatz model [32] is an empirical approach and relies on curve fitting without any physical justification, and was applied to extrusion processes by Keen [35].

**Figure 1:**
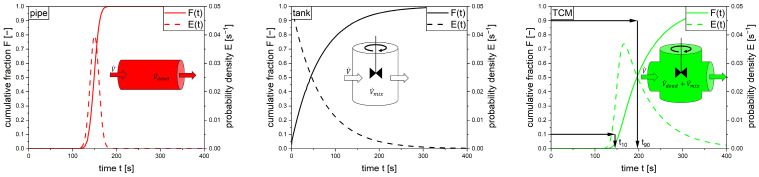
Schematic of modeling approach for the two-compartment model (TCM), consisting of a pipe compartment (**left**) and a tank compartment (**center**). They are convolved to the entire model for residence time distribution (**right**).

The two-compartment model (TCM) by Reitz [28] and the twin dispersion model by Wesholowski [33,36] are preferred in this study based on the specific physical meaning of each parameter, and because they are commonly used in the literature [36,37] for modeling the RTD of extrusion processes. In the twin dispersion model, two single axial dispersion models are coupled mathematically by a convolution considering two hydrodynamic residence times as well as two Bodenstein numbers. This model is able to be fitted to many experimental data and “doubling” of the well-established axial dispersion model facilitates the interpretation of the data and comparison with literature. The “two-compartment model” follows a different concept. There, the extruder is considered to consist of two virtual compartments: an ideal pipe and an ideal continuous stirred-tank reactor. The corresponding residence time models are combined by a convolution as well. There, the residence time in a pipe is described by a Gaussian error function having two parameters, a mean time and a standard deviation (Figure 1, left). Additionally, the residence time in the continuous stirred-tank reactor is described by an exponential decay having just the rate constant (Figure 1, center). Coupling both processes leads to the desired probability density distribution ($E\left(t\right)$) or probability distribution function ($F\left(t\right)$) representing the residence time distribution in the extruder (Figure 1, right).

The meaning of the three model parameters were elucidated by Reitz [28] and attributed to the volume of the pipe ($V_{dead}$), the volume of the continuous stirred-tank ($V_{mix}$) as well as an axial dispersion term in the pipe flow characterized by a coefficient of variation ($\sigma_{dead}^{*}$).

(3) $$E\left(t\right)=\frac{1}{2}\cdot \frac{\dot{V}}{V_{mix}}\cdot \exp\left(\frac{1}{2}\cdot {\left(\frac{\dot{V}}{V_{mix}}\right)}^{2}\cdot {\left(\sigma_{dead}^{*}\cdot \frac{V_{dead}}{\dot{V}}\right)}^{2}-\left(\frac{\dot{V}}{V_{mix}}\right)\left(t-\left(\frac{V_{dead}}{\dot{V}}\right)\right)\right)\cdot erf\left(\frac{\left(\frac{\dot{V}}{V_{mix}}\right)\cdot {\left(\sigma_{dead}^{*}\cdot \frac{V_{dead}}{\dot{V}}\right)}^{2}-\left(t-\left(\frac{V_{dead}}{\dot{V}}\right)\right)}{\sigma_{dead}^{*}\cdot \frac{V_{dead}}{\dot{V}}\cdot \sqrt{2}}\right)$$

So far, all investigations focus on residence time distributions within the entire extrusion process, requiring numerous assumptions and safety margins in formulation design. Following the meaning of quality by design [38], it would be beneficial to look at the temperature exposure as a function of time, which is not fixed but has a certain probability. This insight would be the key in formulation and process development, mitigating the risk of incomplete drug dissolution, thermal degradation and content uniformity issues. Complex fluid dynamics is a valuable tool in this respect, giving insights into the processes within the extruder [39]. However, it is time-consuming to simulate an entire process unit due to its high computational effort. Moreover, in terms of formulation and process design, numerous iterations are required, which is why this technology is not the preferred choice. However, a precise assessment of residence time distribution with respect to the screw length (axially) remains unreported, despite its relevance to pharmaceutical applications. All 1D-modeling approaches require equipment as well as formulation-specific parameters. In the preferred approach of Pawlowski, a pressure and power characteristic is used [16], but residence-time-related parameters for each individual type of screw element are needed.

The aim of this study is to characterize the most common screw elements used in pharmaceutical hot-melt extrusion with respect to residence time. Therefore, an experimental setup will be designed and optimized for reliable determination. A modeling framework will be established to derive element-specific, physically meaningful dimensionless parameters to characterize the residence time behavior of each individual type of screw element.

## 2. Materials and Methods

### 2.1. Materials

Silicone oil (Newtonian fluid, Bluesil FLD 47V100000, Elkem Silicones, Lübeck, Germany) with a viscosity of 96 Pas at 25 °C [40] was utilized as a model compound. Sudan III red (Carl Roth GmbH, Karlsruhe, Germany) served as the tracer substance for residence time measurement and was chosen based on its red color, as well as its miscibility with silicon oil. Stock solutions with silicon oil were prepared with a liquid tracer die.

### 2.2. Extrusion Screw Test Rig Method

**Figure 2:**
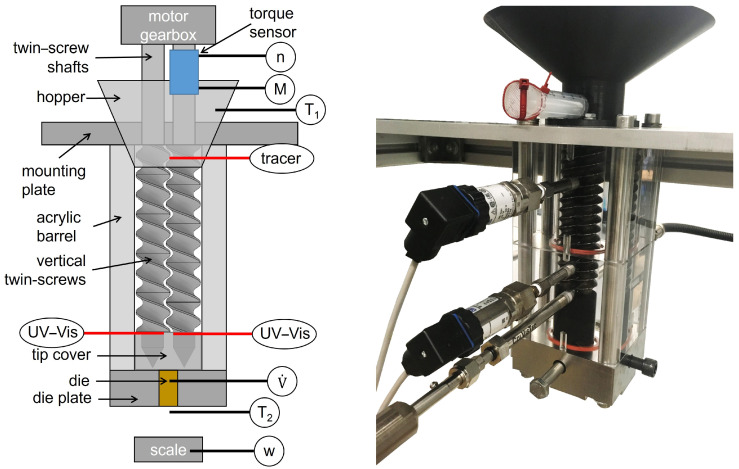
Schematic of vertically arranged twin-screw extruder (**left**) with tracer injection directly in the intermeshing section and measurement directly beyond the screws. Installed setup (**right**).

The individual extrusion screw elements were characterized in a specially designed test rig, which has been used in previous investigations [40,41]. This was geometrically similar to the Leistritz ZSE 27 MAXX extruder (Leistritz, Nürnberg, Germany). The twin-screws were vertically arranged to guarantee complete filling of the barrel, which was required to apply the models. The vertical orientation enabled air bubbles to rise to the top, resulting in a homogeneous, single-phase fluid (Figure 2).

Besides the torque (M), the temperature (T) and the pressure sensors (p), the test rig was additionally equipped by UV-Vis probes (TPMP, ColVisTec, Berlin, Germany), allowing an inline tracer determination via a transmission measurement. The tracer solution was injected via a syringe at the top of the screws. Screw speed (n) as well as die diameters (mm) were altered to adjust the volume flow which was assessed by a scale (w).

### 2.3. Residence Time Measurement Method

For residence time measurements a tracer solution consisting of Sudan III red in silicon oil was injected into the intermeshing region at the top of the screw (Figure 2). The tracer concentration was chosen carefully to meet the appropriate absorbance according to the Lambert–Beer Law. A UV-Vis spectrometer (Inspectro X, ColVisTec, Berlin, Germany) was attached at the bottom of the screws via probes (TPMP, ColVisTec, Berlin, Germany) using fiber optic cables. The distance between the radiating and receiving probes was set to 15 mm and maintained carefully throughout all measurements. Once a day, immediately after startup and equilibration, the spectrometer was calibrated using standardized ceramic tiles (ColVisTec, Berlin, Germany) to scale the absorbance range. The measurements were performed using xenon light flashes of 80 ms while 40 flashes were combined in a single measurement. The wavelength range from 485 to 515 nm was used for calibration and a linear range for absorbance values between 0.2 and 2 was found ($R^{2}$ = 0.998). The residence time measurements were started by injecting the tracer and starting the data acquisition simultaneously using a sampling rate of 2 Hz. The individual spectra were transferred into tracer concentrations, and the concentration time profiles c(t) were utilized for residence time evaluation, as discussed before.

## 3. Results and Discussion

### 3.1. Determination of Residence Times

In pharmaceutical hot-melt extrusion, conveying, as well as kneading, elements are commonly used [42]. That is why three common conveying elements (GFA-20, GFA-30, GFA-40), as well as two kneading (KB-30, KB-60) elements, were used in this study. The kneading element with a 90° staggering angle (KB-90) was not considered since its performance relies on the properties of neighboring elements due to a lack of transport capacity [40]. The characterization of the individual screw elements with respect to residence time was performed using silicon oil with Newtonian flow behavior. The material was chosen for having a similar viscosity at ambient temperature as polymer melts at process conditions.

**Figure 3:**
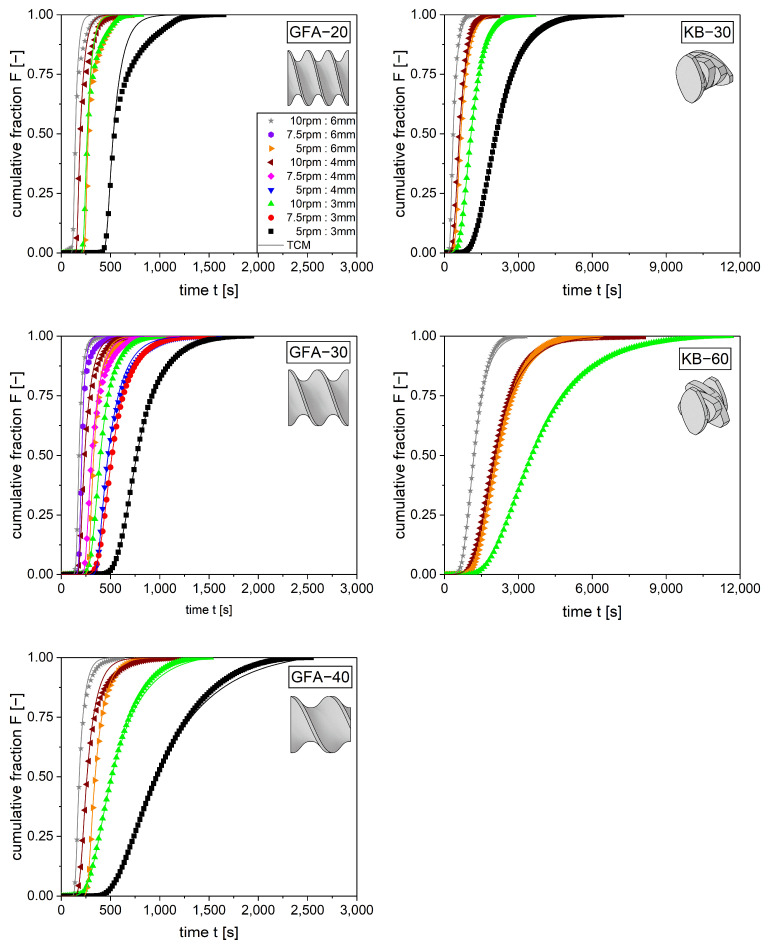
Cumulative residence time distributions F(t) for conveying elements (GFA-20, GFA-30, GFA-40) and kneading elements (KB-30, KB-60) measured at screw speeds of 5, 7.5, and 10 rpm and die diameters of 3, 4, and 6 mm. The solid lines represent the fitted TCM.

**Figure A1:**
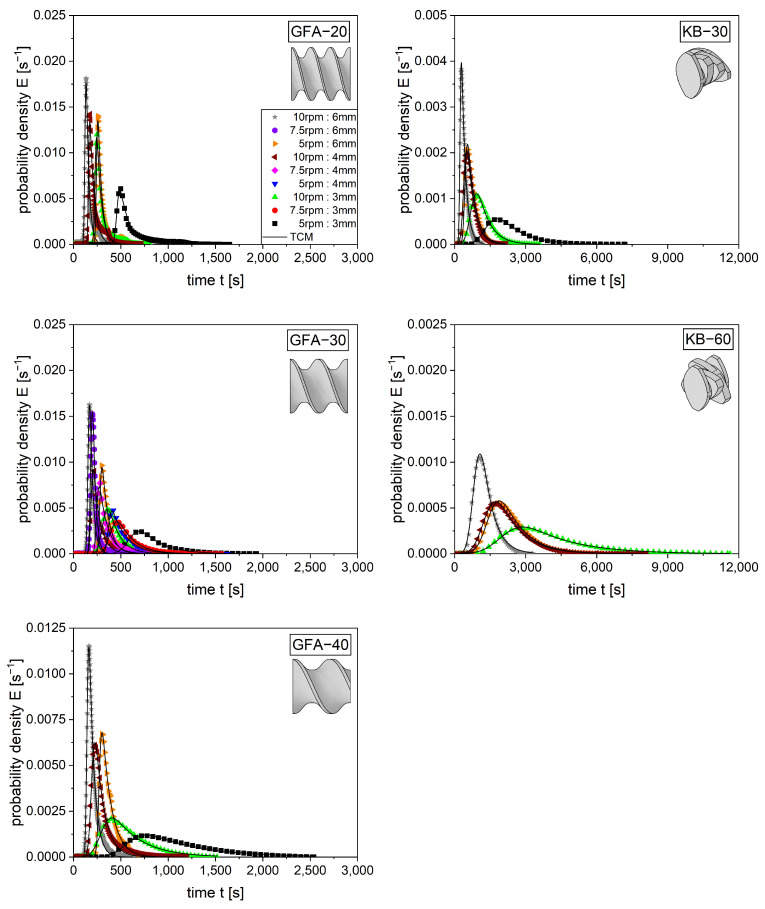
Probability density of residence time distributions E(t) for conveying elements (GFA-20, GFA-30, 225 GFA-40) and kneading elements (KB-30, KB-60) measured at screw speeds of 5, 7.5, and 10 rpm and die diameters of 3, 4, and 6 mm. The solid black lines represent the fitted TCM.

The residence times were determined in a custom-made test rig that has been used to assess the screw parameters (e.g., mixing and transport capacity) before [40,41]. The vertical alignment removes air bubbles from the process, and the acrylic barrel allows visual inspection. One type of screw element was used in this setup at a time to assess the type-specific behavior with respect to residence time. Therefore, a tracer material (a stock solution of Sudan III red in silicon oil) was added into the intermeshing region as a Dirac-Impulse while running the process [43]. Subsequently, the tracer was propagated via the silicon flow axially through the extrusion barrel towards the die, while the tracer concentration was quantified 180 mm from the inlet via an in-line spectrometer acquiring concentration time profiles. These were transferred mathematically into a cumulative residence time distribution (F(t)) (Equation (2)). The results are visualized in Figure 3, while the number of datapoints are reduced and repetitions are not shown for visualization purposes. The results for the volume flow values (Table A1) and the probability density E(t) of the residence time distribution (Figure A1) are given in the appendix.

During the experiments the die diameter, as well as the screw speed, were altered, leading to differences in the dimensionless volume flow and the dimensionless pressure, which are the key parameters in extrusion [44]. The values were chosen based on a previous study [40]. The specific screw parameters regarding pressure and power consumption, as well as volume flow, were also assessed during this study. The results (Table 2) are quite in line with the literature data, giving confidence to the current measurements [40,45,46].

Regarding the conveying elements (GFA-20, GFA-30, GFA-40), the residence time increases with respect to the pitch, which is consistent with the literature [16]. At lower pitch, the material is conveyed more efficiently resulting in higher pressure ($A_{2}$-parameter, Table 2). Moreover, higher volume flows (higher screw speeds, larger die diameters) lead to shorter residence time, since the material volume in the screw (free volume) is fixed, meaning the volume flow correlates inversely with the residence time. The kneading elements (KB-30, KB-60) exhibit much longer residence times than the conveying elements due to their lower conveying capacity. This is indicated by the maximum inherent rate of conveying ($A_{1}$-parameter, Table 2).

### 3.2. Modeling of Residence Times

The residence time in the hot-melt extrusion process is rather complex, and many residence time models have been proposed in the literature for this purpose as previously discussed. Initially, the twin dispersion model from Wesholowski [33] was considered. There, four parameters were used, such as the hydrodynamic residence time and the Bodenstein number, which have an exact physical meaning well accepted in the scientific community. However, closer evaluation with this data set revealed an overfitting by the four fitting parameters, resulting in multiple parameter combinations that described the experimental data quite well. Based on this drawback, the two-compartment model of Reitz [28] was utilized to consider just three fitting parameters: the volume of an imaginary stirred-tank ($V_{mix}$), the volume of an ideal pipe ($V_{dead}$) and the coefficient of variation characterizing the axial mixing in a pipe ($\sigma_{dead}^{*}$) in Equation (3). The Reitz model was fitted to the experimental data (Figure 3) and is denoted by the solid lines. Some systematic deviations were observed for particularly short residence times at the upper quarter of the residence time distribution function (Figure 3, GFA20, black symbols and line). These were attributed to an imperfection of the experimental setup, since it was not possible to apply the viscous tracer solution in an infinitely short Dirac-Impulse as assumed by the model. Neither was it possible to initialize the tracer over the free cross-sectional area (cross-sectional area of the barrel bore not occupied by screws) of the screw homogeneously. Based on this, these deviations were assumed to be an experimental limitation, and no effort was made to incorporate these in the model. Besides this fact, the two-compartment model of Reitz describes the experimental data quite well, which is why it was used for further evaluation.

**Figure 4:**
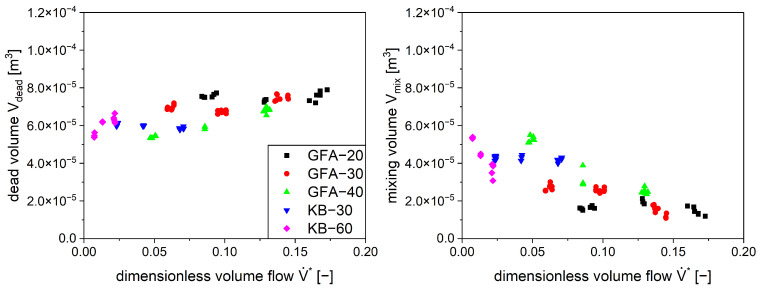
Dead volume ($V_{dead}$) (**left**) and mix volume $\left(V_{mix}\right)$ (**right**) as a function of dimensionless volume flow (${\dot{V}}^{*}$) for conveying elements (GFA-20, GFA-30, GFA-40) and kneading elements (KB-30, KB-60) generated out of fitted parameters by the TCM.

Further investigations dealt with the evaluation of the two-compartment model parameters ($V_{dead}$, $V_{mix}$, $\sigma_{dead}^{*}$), which were plotted with respect to the dimensionless volume flow (Figure 4). The dimensionless volume flow is defined as the ratio of the volume flow ($\dot{V}$) to the product of the cubed barrel diameter (d) and screw rotation speed (n).

(4) $${\dot{V}}^{*}=\frac{\dot{V}}{n\cdot d^{3}}$$

This served as the reference in various evaluations regarding screw elements and was utilized in this study as well [40,41,45]. The mixing volume as well as the dead volume are independent from the dimensionless volume flow, and a regression analysis revealed no significant (α = 0.05) difference in the slope to “0” for all elements besides GFA-40. There might be a change in mixing or transport regime for this element at high throughputs, which was not investigated further and ignored for generalization purposes.

Adding the mixing volume ($V_{mix}$) and dead volume ($V_{dead}$) led to a grouping of the data at distinctive levels for the conveying elements on one hand and the kneading elements on the other hand.

(5) $$V_{overall}=V_{dead}+V_{mix}\equiv V_{free}$$

**Figure 5:**
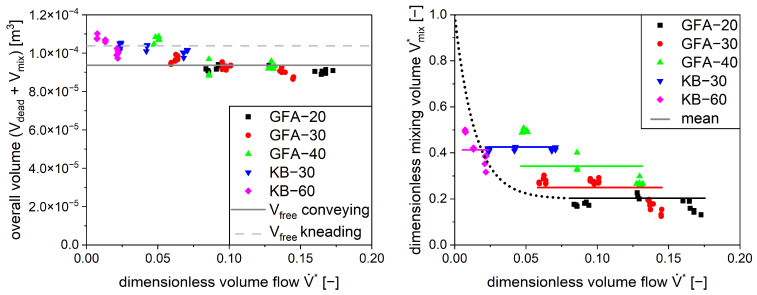
Overall volume ($V_{overall}$) (**left**) with horizonal lines reflecting the free volume ($V_{free}$) of the conveying and the kneading elements calculated from the screw geometry. Dimensionless mixing volume ($V_{mix}^{*}$) (**right**) with horizontal lines reflecting the mean values ($\bar{V_{mix}^{*}}$) for each screw element. The theoretical mixing behavior of ideal stirred-tank as dotted line, exemplary for GFA-20. Both figures capture conveying elements (GFA-20, GFA-30, GFA-40) and kneading elements (KB-30, KB-60) as a function of dimensionless volume flow (${\dot{V}}^{*}$).

These values correspond to the free volume ($V_{free}$, volume in the barrel bore not occupied by the screw) of the screw elements, which can be derived from the screw geometry (Figure 5, left, solid line). Due to the axial gaps within the kneading elements, the free volume values are slightly higher compared to the conveying elements. Based on this, the free volume of the individual screw element was implemented in the two-compartment model through a new “mixing parameter” ($V_{mix}^{*}$), given as the ratio of the mixing volume and free volume ($V_{free}$).

(6) $$V_{mix}^{*}=\frac{V_{mix}}{V_{free}}$$

A mixing parameter of 0 indicates ideal pipe behavior, where the transport is dominating and just a little mixing occurs based on axial dispersion. A mixing parameter of 1, on the other hand, corresponds to continuous stirred-tank reactor performance, where the mixing is dominating over the transport. Based on this, the two-compartment model (Equation (3)) was modified by replacing the dead volume and the mixing volume via Equations (5) and (6), respectively. This allows the experimental data to be fitted using just two parameters ($V_{mix}^{*}$, $\sigma_{dead}^{*}$).

Even if the new mixing parameter was not independent of the dimensionless volume flow, it was impossible to identify a mathematical correlation based on the variability in the data. Theoretically, this function should have a mixing parameter of 1 at a volume flow of “0”, and at high volume flows, it should converge toward a fixed value as illustrated by the dotted function in Figure 5 (right) (GFA-20). This kind of function might have two parameters: a rate constant (k) as well as an equilibrium constant. However, it was unfeasible to fit these two parameters to the experimental data, so the arithmetic mean of the mixing parameter ($\bar{V_{mix}^{*}}$) was used for the individual screw elements.

**Figure 6:**
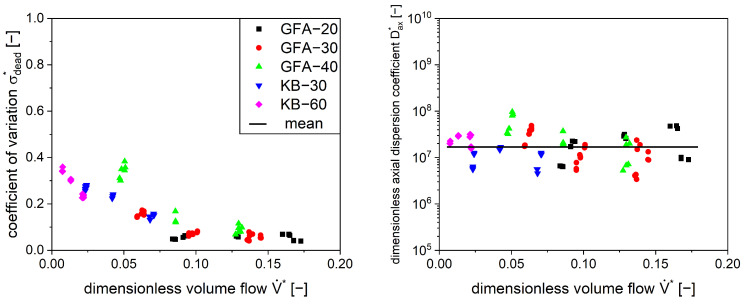
Coefficient of variation ($\sigma_{dead}^{*}$) (**left**) and dimensionless dispersion coefficient ($D_{ax}^{*}$) (**right**). Both figures capture conveying elements (GFA-20, GFA-30, GFA-40) and kneading elements (KB-30, KB-60) as a function of dimensionless volume flow (${\dot{V}}^{*}$). The horizontal line (**right**) reflects the mean value ($\bar{D_{ax}^{*}}$) over all screw elements.

The two-compartment model considers the axial dispersion as being related to the standard deviation, which was normalized to the hydrodynamic residence time derived from the dead volume and the volume flow, giving a coefficient of variation ($\sigma_{dead}^{*}$, Figure 6 left). This was independent from the screw element used, but clearly a function of the dimensionless volume flow. Lower volume flows lead to longer residence times and more time for axial dispersion and a larger coefficient of variation. The coefficient of variation was transformed into a Bodenstein number (Bo), which is more commonly used in the field of process engineering rather than the coefficient of variation. Therefore, a reactor with two open ends was considered while the following equation was applied [47]:

(7) $${\left(\sigma_{dead}^{*}\right)}^{2}=\frac{\sigma^{2}}{{\bar{t}}^{2}}=\frac{2}{Bo}+\frac{8}{Bo^{2}}$$

Solving for the Bodenstein number leads to:

(8) $$Bo=\frac{1+\sqrt{1+8\cdot {\sigma_{dead}^{*}}^{2}}}{{\sigma_{dead}^{*}}^{2}}$$

After this transformation, the Bodenstein number is still a function of the dimensionless volume flow. Therefore, the screw geometry (diameter, pitch, length) as well as the volume flow were used to account for equipment and process attributes. The axial dispersion coefficient ($D_{ax}$) was derived assuming an ideal pipe, considering the mean flow velocity (u) and the pipe length (l) [22].

(9) $$D_{ax}=\frac{u\cdot l}{Bo}$$

However, based on the different screw geometries, these two parameters are altered accordingly. The pipe length was considered to be equal to the flight length ($l_{fl}$) since the material is following the groove in the screw and is traveling a longer distance than the length of the screw element. In terms of the conveying elements, the flight length was derived from the screw outer diameter ($d_{a}$) and the element length ($l_{el}$), as well as the pitch ($l_{p}$), using the Pythagorean theorem. The apparent flight length of the kneading elements was calculated similarly using the staggering angle as a stepwise change in pitch.

(10) $$l_{fl}=\frac{l_{el}}{l_{p}}\sqrt{{\left(\pi \cdot d_{a}\right)}^{2}+{l_{p}}^{2}}$$

Since the free volume of a screw element is conserved, a change in groove length will alter the free cross-sectional area ($A_{free}$). Therefore, a distinction was made between the free cross-sectional area normal to the axial direction as used before and the free cross-sectional area normal to the material flow. The latter is relevant for calculation of the mean material velocity based on volume flow, flight length and free volume of the screw.

(11) $$u=\frac{\dot{V}}{A_{free}}=\frac{\dot{V}\cdot l_{fl}}{V_{free}}$$

Using Equations (8)–(11), the axial dispersion coefficient was calculated from the Bodenstein number and the screw geometry, as well as the volume flow. This parameter was independent from screw element type and dimensionless volume flow.

Unfortunately, the axial dispersion coefficient is not dimensionless and is highly related to material attributes like viscosity (η) of the polymer as well as size (radius (r)) of the tracer molecule. Therefore, the axial dispersion coefficient ($D_{ax}$) was normalized ($D_{ax}^{*}$) to molecular diffusion (D), as in the Taylor dispersion [48,49].

(12) $$D_{ax}^{*}=\frac{D_{ax}}{D}$$

Therefore, the Stokes-Einstein correlation was applied using the Boltzmann constant ($k_{B}=1.38\cdot 10^{-23}J/K$) [50] and the temperature-corrected viscosity of the silicon oil (η= 96 Pa·s), where the temperature correction was applied by the temperature shift factor ($a_{T}$) [40].

(13) $$D=\frac{k_{B}\cdot T}{6\cdot \pi \cdot \eta \cdot a_{T}\cdot r}$$

The molecular radius (r) of the tracer was calculated from its density (ρ= 1200 kg/m^3^) [51] and its molecular weight ($M_{W}$) yielding a molecular radius of r= 0.488 nm.

(14) $$r=\frac{1}{2}\cdot \sqrt[{3}]{\frac{6\cdot M_{W}}{\pi \cdot \rho \cdot k_{B}}}$$

The dimensionless axial dispersion coefficient is independent from the dimensionless volume flow in the extruder, and rather independent from the screw element used (Figure 6, right). Therefore, an arithmetic mean over all dimensionless volume flows and screw element types was considered ($\bar{D_{ax}^{*}}$). It can be seen as an inherent equipment parameter.

### 3.3. Predicting Residence Times

So far, experimental data have been used to identify the influence of different screw element types on the residence time distribution. Two relevant parameters were derived where $\bar{V_{mix}^{*}}$ is screw-element-type-specific, while $\bar{D_{ax}^{*}}$ is a constant.

**Figure 7:**
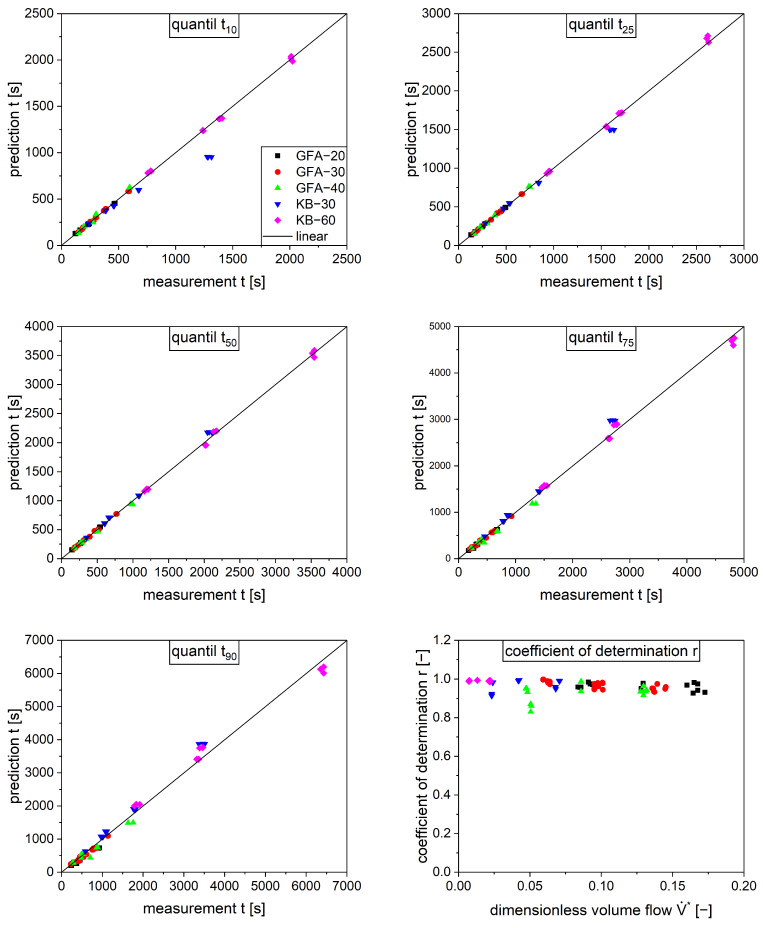
Parity plots comparing predicted and experimentally measured quantiles ($t_{10},t_{25},t_{50},t_{75},t_{90}$) of the residence time distribution for conveying elements (GFA-20, GFA-30, GFA-40) and kneading elements (KB-30, KB-60). The solid line represents perfect agreement between prediction and measurement. The correlation coefficient r as function of dimensionless volume flow as visualized quality measure of the prediction (bottom right).

Based on this observation, residence time distributions were predicted and compared to the original experimental data. To illustrate the large volume of data, parity plots of common quantiles were selected, in which the prediction is plotted as a function of the experimental measurement (Figure 7).

All data points for all quantiles are in close proximity to the bisecting line, confirming the validity of the proposed concept. The coefficient of determination was larger than 0.83, which proves a good agreement of the model with the data.

### 3.4. Application of Proposed Residence Time Model

This section is dedicated to the use of the proposed residence time model to highlight its value for the scientific community. Therefore, an artificial extrusion process is assumed and the influence of screw type to process attributes such as onset and offset of the distribution function, as well as the mixing capacity, is discussed. Thus, a twin-screw extrusion process of an overall length of 20 d (20 times screw diameter) is chosen. The extrusion screw consists of a single type of screw element, while a Newtonian flow behavior (η = 96 Pa·s) is assumed. The screw elements are completely filled with material, and the volume flow is set to predefined values. A more realistic process can be achieved by 1D numerical simulation, but that is beyond the scope of this article; therefore this simplification was made.

**Figure 8:**
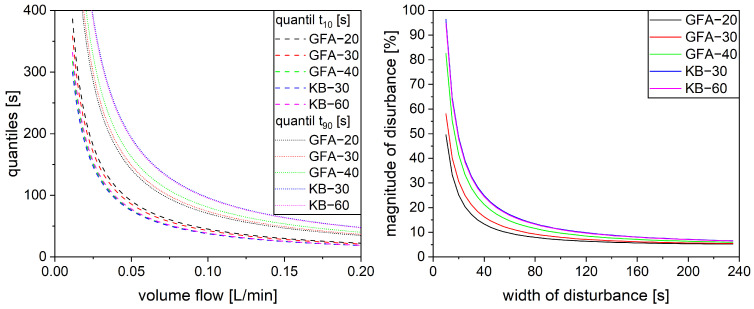
(**Left**): Residence time distribution width $\left(t_{10}\right)$ and $\left(t_{90}\right)$ as a function of volume flow for conveying elements (GFA-20, GFA-30, GFA-40) and kneading elements (KB-30, KB-60). (**Right**): Funnel plot depicting the detectable magnitude of disturbance [%] as a function of disturbance width [s] flow for conveying elements (GFA-20, GFA-30, GFA-40) and kneading elements (KB-30, KB-60). The curves reflect a resulting 5% disturbance on the residence time distribution.

The residence time distribution in hot-melt extrusion is commonly used to tailor the temperature–time profile in preparation of amorphous solid dispersions as discussed before. Therefore, the onset (e.g., $t_{10}$) is correlated with residual drug crystals while the offset (e.g., $t_{90}$) is related to undesired thermal degradation. The residence time distribution functions for different screw elements were calculated (Equations (3), (7) and (12)) and the two quantiles were determined (Figure 8).

Overall, the residence time, as well as the width of its distribution, increases with a decrease in volume flow (Figure 8, left) based on the definition of the hydrodynamic residence time. Having time-dependent processes such as drug dissolution or thermal degradation in the production of amorphous solid dispersions, the volume flow is likely to be a critical quality attribute. An increase in pitch of the conveying elements and the use of kneading elements reduces the dead volume and lowers the onset of the distribution function. In this way the material moves more axially and spirals less than in the grooves of the low pitch of the screw. Therefore, kneading elements show a lower onset than conveying elements, which seems counterintuitive. The width of the distribution (difference between onset $\left(t_{10}\right)$ and offset $\left(t_{90}\right)$) is mainly attributed to the mixing volume increasing with the pitch of the conveying and even more for the kneading elements. When producing amorphous solid dispersion, the goal is to achieve narrow residence time distributions to minimize the risk of thermal degradation. In this respect, conveying elements with low pitch are preferred.

However, the width of the residence time distribution is also utilized to assess the axial mixing capacity of a continuous process such as hot-melt extrusion. Wide residence time distributions equalize fluctuations in material composition, improving content uniformity. Both the magnitude of the disturbance and its duration need to be considered, which is why a characteristic “funnel plot” is commonly utilized [23] (Figure 8, right). Considering the remaining fluctuation of 5% in accordance with the European Pharmacopeia 2.9.40 [52], characteristic functions for the individual screw elements could be found at a dimensionless volume flow of 0.2. For these evaluations, a step function was generated, emulating a disturbance characterized by a step height (magnitude, percent deviation from set) and a step width (duration) [53]. This was convolved with the residence time distribution which was dampening the magnitude of the disturbance, which was assessed subsequently [54]. The function in the corresponding funnel plot separates regions of disturbances greater than the specification (above the function) and disturbances below the specification (underneath the function).

For all geometries, large disturbances for a short time period, and low disturbances for a longer time period, are acceptable in order to meet the pharmacopeia specification (Figure 8, right). With respect to the mixing capacity, kneading elements are preferred over conveying elements, and larger pitch is more sufficient within the conveying elements. This behavior is attributed to the mixing capacity of the individual screw elements quantified by the mixing volume ($\bar{V_{mix}^{*}})$ in Table 3. This gives an inverted sequence of preferred screw types compared to the previous paragraph considering thermal degradation. Finally, the residence time and its modeling are valuable tools to mediate conflicting specifications such as thermal degradation and content uniformity.

## 4. Conclusions

This study focuses on the effect of different screw-element types on the residence time and its distribution in pharmaceutical hot-melt extrusion processes. Therefore, common conveying and kneading elements were considered.

A test rig as well as an analytical method were designed and optimized for reliable experimental determination of the residence time for various screw types. The screw speed together with the die diameter were altered systematically to assess the screw type performance.

The two-compartment model of Reitz was utilized to describe the behavior of different screw types mathematically using three model parameters. Based on experimental data using the equipment geometry, as well as process conditions, two new equipment- and material-independent parameters were derived to quantify screw-element-type behavior.

The kneading elements exhibited higher dimensionless mixing volumes ($\bar{V_{mix}^{*}}$) and broader RTDs compared to conveying elements, which reflects their superior mixing capability. The dimensionless axial dispersion coefficient ($\bar{D_{ax}^{*}}$) showed no systematic dependence on element geometry or volume flow and was therefore treated as an equipment-specific parameter.

The value of this modeling approach was demonstrated by subsequently connecting the findings to production processes for amorphous solid dispersions. There, the value of the onset and the offset of the residence time distribution function were discussed, as well as the influence on mixing capacity using the well-known funnel plot. These findings provide a quantitative basis for screw design and process control in hot-melt extrusion, enabling targeted selection of screw elements to balance mixing performance and disturbance sensitivity for specific pharmaceutical applications.

## Figures and Tables

**Table 1:** Overview for RTD models in the literature, listing number of model parameters.

Model	Author	Year	Number of Parameters
Tank in series	MacMullin and Weber [30]	1935	2
Leakage flow	Janssen [31]	1979	4
Axial dispersion	Levenspiel [22]	1999	2
Zusatz model	Zusatz [32]	2001	3
Two-compartment	Reitz [28]	2013	3
Twin dispersion	Wesholowski [33]	2018	4

**Table 2:** Dimensionless screw parameters of conveying and kneading elements for Newtonian flow behavior as pressure characteristics ($A_{1},A_{2}$) [16] and power characteristics ($B_{1},B_{2}$) [16] by linear regression and the corresponding confidence interval. $\bar{x}$ ± CI (α = 0.05).

	Conveying Elements			Kneading Elements	
Screw	GFA-20	GFA-30	GFA-40	KB-30	KB-60
$A_{1}$	0.217 ±0.043	0.324 ±0.033	0.419 ±0.098	0.323 ±0.177	0.062 ±0.011
$A_{2}$	4304 ±288	2792 ±64	1850 ±68	819 ±41	239 ±38
$B_{1}$	0.426 ±0.224	0.704 ±0.388	0.973 ±0.150	1.540 ± 0.245	9.337 ±1.015
$B_{2}$	4039 ±360	3056 ±147	2673 ±137	1862 ±37	1384 ±33

**Table 3:** Parameters for prediction of the residence time distribution for conveying and kneading elements. Mean dimensionless mixing volume ($\bar{V_{mix}^{*}}$) for conveying elements (GFA-20, GFA-30, GFA-40) and kneading elements (KB-30, KB-60) by arithmetic mean and the confidence interval: $\bar{x}$ ± CI (α = 0.05). Mean normalized axial dispersion coefficient ($\bar{D_{ax}^{*}}$) for all screw elements.

	Conveying Element			Kneading Element	
Screw	GFA-20	GFA-30	GFA-40	KB-30	KB-60
$\bar{V_{mix}^{*}}$	0.2031 ±0.0491	0.2493 ±0.0696	0.3426 ±0.1281	0.4254 ±0.0306	0.4135 ±0.0684
$\bar{D_{ax}^{*}}$	$2.15\cdot 10^{7}\pm 3.64\cdot 10^{7}$				

## Data Availability

The raw data supporting the conclusions of this article are included in the Supplementary Materials.

## Supplementary Materials

The following supporting information can be downloaded at: https://www.mdpi.com/article/10.3390/pharmaceutics18070883/s1.

## Abbreviations

The following abbreviations and symbols are used in this manuscript:

Latin Symbols	definition	unit
$A_{1},A_{2}$	dimensionless pressure parameters	−
$B_{1},B_{2}$	dimensionless power parameters	−
$A_{free}$	free cross-sectional area	$m^{2}$
$a_{T}$	temperature shift factor	−
Bo	Bodenstein number	−
$c\left(t\right)$	concentration function	$mol\cdot L^{-1}$
D	molecular diffusion	$m^{2}\cdot s^{-1}$
$D_{ax}$	axial dispersion coefficient	$m^{2}\cdot s^{-1}$
$D_{ax}^{*}$	dimensionless axial dispersion coefficient	−
$\bar{D_{ax}^{*}}$	mean dimensionless axial dispersion coefficient	−
d	barrel diameter	m
$d_{a}$	screw outer diameter	m
dt	differential of time	s
$E_{A}$	Arrhenius activation energy	$J\cdot {mol}^{-1}$
$E\left(t\right)$	probability density E	$s^{-1}$
$F\left(t\right)$	cumulative fraction	−
$k_{B}$	Boltzmann constant	$J\cdot K^{-1}$
l	axial length	m
$l_{fl}$	flight length	m
$l_{el}$	element length	m
$l_{p}$	pitch	m
$M_{W}$	molecular weight	$kg\cdot {mol}^{-1}$
n	screw speed	$s^{-1}$
r	molecular radius	m
T	Temperature	K
t	time	s
$\bar{t}$	mean residence time	s
u	velocity	$m\cdot s^{-1}$
$\dot{V}$	volume flow	$m^{3}\cdot s^{-1}$
${\dot{V}}^{*}$	dimensionless volume flow	−
$V_{dead}$	volume of the pipe/dead volume	$m^{3}$
$V_{mix}$	volume of the tank/mixing volume	$m^{3}$
$V_{overall}$	overall volume	$m^{3}$
$V_{free}$	free volume between the screws	$m^{3}$
$V_{mix}^{*}$	dimensionless mixing volume	−
$\bar{V_{mix}^{*}}$	mean dimensionless mixing volume	−
Greek symbols	definition	unit
η	viscosity	Pa·s
π	dimensionless constant	−
ρ	density	$kg\cdot m^{-3}$
$\sigma_{dead}^{*}$	dimensionless coefficient of variation	−
${\sigma_{dead}^{*}}^{2}$	dimensionless variance	−
$\sigma^{2}$	variance	s
Abbreviations	definition	
HME	hot-melt extrusion	
TCM	two-compartment model	

## Appendix A

**Table A1:** Volume flow values for the corresponding residence time distribution experiments, measured in the vertically arranged twin-screw extruder.

Screw	Screw Speed [rpm]	Die Diameter [mm]	Volume Flow [mL/min]
GFA-20	10	6	35.51
GFA-20	5	6	19.32
GFA-20	10	4	27.30
GFA-20	10	3	20.31
GFA-20	5	3	9.81
GFA-30	10	6	29.67
GFA-30	75	6	24.34
GFA-30	5	6	15.70
GFA-30	10	4	21.80
GFA-30	75	4	16.38
GFA-30	5	4	11.04
GFA-30	10	3	13.86
GFA-30	75	3	10.45
GFA-30	5	3	6.88
GFA-40	10	6	28.47
GFA-40	5	6	14.88
GFA-40	10	4	18.56
GFA-40	10	3	10.95
GFA-40	5	3	5.51
KB-30	10	6	15.24
KB-30	5	6	7.84
KB-30	10	4	9.16
KB-30	10	3	5.20
KB-30	5	3	2.69
KB-60	10	6	4.58
KB-60	5	6	2.55
KB-60	10	4	2.85
KB-60	10	3	1.64
